# Hepatocellular Carcinoma Intrinsic Cell Death Regulates Immune Response and Prognosis

**DOI:** 10.3389/fonc.2022.897703

**Published:** 2022-07-07

**Authors:** Valli De Re, Anna Rossetto, Alessandro Rosignoli, Elena Muraro, Vito Racanelli, Maria Lina Tornesello, Aron Zompicchiatti, Alessandro Uzzau

**Affiliations:** ^1^ Immunopatologia e Biomarcatori Oncologici/Bio-proteomics Facility, Centro di Riferimento Oncologico di Aviano (CRO), IRCCS, Aviano, Italy; ^2^ General Surgery, Azienda Sanitaria Universitaria Friuli Centrale (ASUFC), San Daniele del Friuli, Udine, Italy; ^3^ Program of Hepatobiliopancreatic Surgery, Azienda Sanitaria Universitaria Friuli Centrale (ASU FC), University of Udine, Udine, Italy; ^4^ Department of Interdisciplinary Medicine, Medical School, Aldo Moro University of Bari, Bari, Italy; ^5^ Molecular Biology and Viral Oncology Unit, Istituto Nazionale Tumori IRCCS Fondazione G. Pascale, Napoli, Italy

**Keywords:** hepatocellular carcinoma, cell death, necrosis, immune response, ablation, prognosis

## Abstract

Ablative and locoregional treatment options, such as radiofrequency, ethanol injection, microwave, and cryoablation, as well as irreversible electroporation, are effective therapies for early-stage hepatocellular carcinoma (HCC). Hepatocyte death caused by ablative procedures is known to increase the release of tumor-associated antigen, thus enhancing tumor immunogenicity. In addition, the heat ablative resection induces pyroptotic cell death accompanied by the release of several inflammatory factors and immune-related proteins, including damage-associated molecular patterns (DAMPs), heat shock proteins (HSPs), ficolin 3, ATP, and DNA/RNA, which potentiate the antitumoral immune response. Surgical approaches that enhance tumor necrosis and reduce hypoxia in the residual liver parenchyma have been shown to increase the disease-free survival rate by reducing the host’s immunosuppressive response. Scalpel devices and targeted surgical approach combined with immune-modulating drugs are an interesting and promising area to maximize therapeutic outcomes after HCC ablation.

## Introduction

The incidence of hepatocellular carcinoma (HCC) rises worldwide, despite the enormous progress made by the introduction of hepatitis B virus (HBV) vaccine and antiviral therapy for hepatitis C virus (HCV).

Therapeutic chances are still limited due to late diagnosis with impossibility of radical intent and limited treatment options.

In recent years, guidelines have been developed to standardize care and resources and for therapeutic optimization. However, the most recent therapeutic classification, according to tumor stages and the best-expected benefit, the Barcelona Clinic Liver Cancer (BCLC) (1), has already undergone modifications due to the complexity of the disease. Indeed, it is difficult to have a guideline algorithm that includes all the possible clinical presentations of HCC.

Therapeutic options for HCC at an early stage (BCLC 0/A) are suitable for radical therapies (surgical resection, transplantation, and locoregional ablation), all of which are potentially curative. The decision for surgical resection requires consideration of the patient’s residual liver function, transplantation availability of liver organ, and locoregional therapies of the tumor size and the number and location of the neoplastic nodules. Current evidence, reviewed by Haber et al. (2), supports the effective development of immunotherapies, which can target potential micrometastases, to prevent recurrence after these curative approaches, although there is high heterogeneity in the patient’s survival time due to the very complex interaction between the immune response and the tumor microenvironment (TME).

When altered/infected cells die, death modalities activate specific physiological signaling that triggers the inflammatory process and the consequent host’s immune response while avoiding tissue damage for a long period.

There are distinct types of cell death depending on the stimulus received by the cell, and this can result to a difference in the induction of the antitumor immune response (3).

The mechanisms of cell death can be divided into three main types: apoptosis, autophagy, and necrosis (**Table 1**).

**Table 1:** Major differences among apoptosis, autophagy, and necrosis.

	Apoptosis	Autophagy	Necrosis
			
The primary trigger of cell death	Trauma, aging, cellular stress, cell renewal, inflammation, pathogens	Nutrient deprivation, hypoxia	Trauma, infection, inflammation
Nucleus	Marked chromatin condensation, programmed nuclear fragmentation	Minor change	Minor chromatin condensation, random nuclear degradation
Mitochondrial and cell swelling	±, release cytochrome C, bcl2, and caspase cascade	±	Yes, failure ATP production, ROS production, AIF release
Cytoplasmatic vacuoles	Minor change	Yes, organelle degradation	Swelling
Caspase 3 activity	Yes	No	No
Caspase 1 activity	No	No	Yes, the pyroptosis subtype
RIP kinase	No	No	Yes
Heat shock proteins	Calreticulin	Yes	Yes
Phosphatidylserine	Yes	Yes	No
Cathepsin B, lysosomal activity	No	Yes	No
Loss of membrane integrity	No loss integrity,apoptotic bodies	No loss integrity,autophagic vacuoles	Yes, loss membrane integrity, inflammatory and cytokine content release
Response	Anti- and pro-inflammatory	Anti-inflammatory	Pro-inflammatory, affects neighboring cells

RIP, Receptor-interacting serine/threonine-protein kinase; bcl-2, antiapoptotic B-cell lymphoma 2; ATP, adenosine triphosphate; ROS, Reactive oxygen species; AIF, Apoptosis-Inducing Factor.

### Apoptosis

Apoptosis, also called “programmed cell death,” produces apoptotic bodies and is characterized by the exposure of calreticulin on the outer layer membrane. Macrophages engulf and digest the apoptotic bodies (efferocytosis), thus resulting in minor tissue destruction and inflammatory response than other types of cell death. Apoptosis normally occurs during development and aging, and, in some pathological conditions resulting from infections or cell damage, it is activated by intracellular signaling pathways involving a proteolytic caspase cascade.

Apoptosis is the most important defense mechanism against hepatocarcinogenesis. The liver has an incredible regenerative capacity that makes it unique; normal hepatocyte turnover consists of a small number of dead cells (0.05%) by apoptosis and is associated with low serum levels of both liver enzymes alanine aminotransferase (ALT) and aspartate aminotransferase (AST), whose levels increase with the number of dead cells. The activity of some cancer drugs, such as anthracyclines, is based precisely on the modulation of apoptosis to elicit the stimulus necessary to induce cell death (4, 5).

There are two main pathways for apoptosis: the intrinsic and the extrinsic pathways.

The intrinsic pathway is triggered by stress signals induced by DNA damage and activation of the *P53* pathway. The extrinsic pathway is initiated by an external ligand [e.g., tumor necrosis factor-α (TNF-α), Fas] binding to a death receptor [e.g., Fas (CD95L), tumor necrosis factor receptor 1 (TNFR-1)] and results mainly from the activation of the immune response against the altered cells.

**Figure 1:**
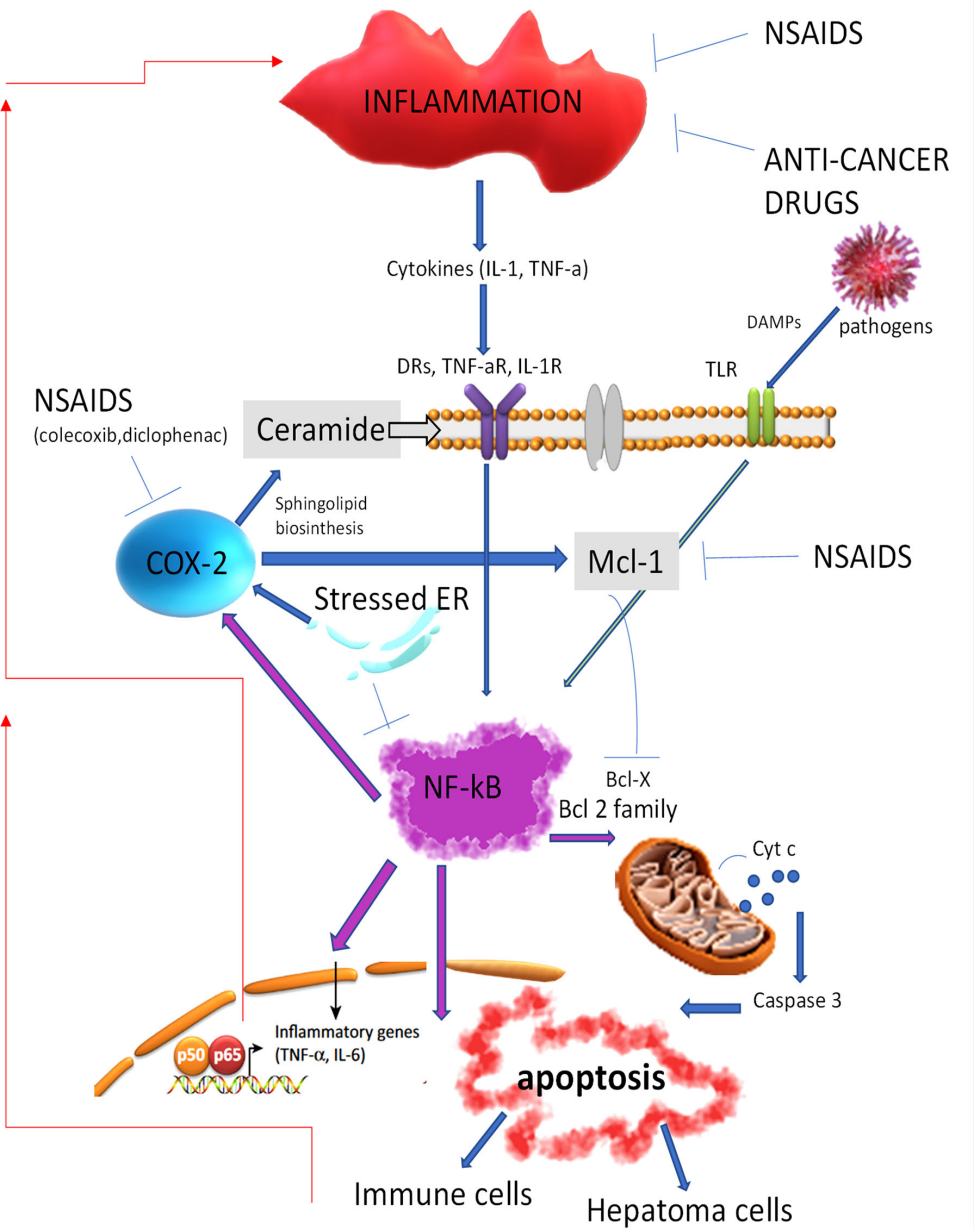
Non-steroidal anti-inflammatory drugs and anticancer drugs by inhibiting inflammation reduce the activation of the NF-kB signaling, resulting in the apoptosis cell death and the activation of the immune cell response. DRs, death receptors; NSAIDs, non-steroidal anti-inflammatory drugs; NF-kB, nuclear factor-kB; BCL-2, antiapoptotic B-cell lymphoma; Cyt c, cytochrome C. TNF-aR, Tumor necrosis factor receptor alfa; IL-1R, Interleukin-1 receptor; DAMPs, Damage-associated molecular patterns; TLR, Toll like receptor; NSAIDS, Non-steroidal anti-inflammatory drugs; COX-2, Cyclooxygenase-2; Mcl-1, Induced myeloid leukemia cell differentiation protein; ER, endoplasmic reticulum; Bcl-x, Apoptosis regulator Bcl-extra; IL-6, Interleukin 6.

Most of the genetic alterations observed in HCC lead to high levels of inhibitors of the proapoptotic *BCL-2* family members (i.e., *BCL-2*, *BAX*, *BAK*) and the permeabilization of the mitochondrial membrane with the release of cytochrome c that activates caspases 8 and 3 (6). It is well recognized that the nuclear factor-κB (*NF-κB*) pathway, involved in inflammation and immunity, could also regulate apoptosis, often by regulating *BCL-2* expression (7). An activation of the *NF-κB* pathway might be generated through different mechanisms, such as activation of the Toll-like receptors (TLRs) by pathogens or the release of inflammatory cytokines in the microenvironment [e.g., TNF and interleukin-1 (IL-1)], and it is hypothesized that HCC is linked to an uncontrolled or dysfunctional inflammatory/immune response. This evidence is supported by the observations that administration of non-steroidal anti-inflammatory drugs (NSAIDs), such as aspirin, reduces the incidence and mortality of gastrointestinal malignancies (8, 9). A direct relationship between the immune system’s response and NSAIDs in HCC has been demonstrated, such as NSAIDs that downregulate *Mcl-1* expression, thus inhibiting the translocation of the antiapoptotic *Bax* in the mitochondria. Also, celecoxib, an NSAIDs inhibitor selective for cyclooxygenase-2 (COX-2), leads to the production of ceramide, a sphingolipid metabolite that induces apoptosis by consolidating the lipid raft in the cytoplasmatic membrane necessary for the clustering and activation of death receptors (10) (**Figure 1**). Moreover, chemotherapeutic drugs, i.e., oxaliplatin and anthracyclines, or ionizing radiation that induces apoptosis showed a superior clinical response when combined with NSAIDs.

### Autophagy

Autophagy recycles nutrients, remodels, and discards unwanted cytoplasmatic constituents through a lysosome-dependent catabolic process to balance energy and survive stressful conditions such as hypoxia, nutrient deficiency, or chemotherapy. Autophagy is classified into macroautophagy, microautophagy, and chaperone-mediated autophagy (CMA). In macroautophagy, autophagosomes are composed of double-membrane vesicles and entrapped cytosolic components and organelles, then by fusion with lysosomes, they form the autolysosome that degrades the sequestered components, making them again available for cell metabolism (11). The specific removal of aggregated proteins and damaged mitochondria that produce high levels of mitochondrial reactive oxygen species (ROS) is called “mitophagy.” In microautophagy, cytosolic components are directly invaginated into the lysosomes. In CMA, invagination into the lysosomal lumen is selectively mediated by the formation of protein complexed with chaperone proteins that are sequestered by special lysosomal membrane receptors.

Though autophagy is considered a self-homeostatic strategy, it is also considered a cell death process, as excessive autophagy can lead to self-eating, but contrary to apoptosis, it is considered an immunogenic cell death. Indeed, autophagy, by degrading intracellular pathogens and undesirable cytoplasmatic constituents, can deliver endogenous antigens to Major Histocompatibility Complex (MHC) molecules, thus allowing antigen presentation to immune cells (12). However, in the first phase of a tumor development, autophagy activation is closely linked to a benefit in cancer cell survival in a hypoxic and nutrient-exhausted TME, and immune cells perform either protumor or antitumor functions depending on the signals they receive from the TME. Specifically, tumor cells show an increase in arginine and glucose uptake necessary for their increased protein biosynthesis and energy source, which leads to a reduction of arginine and an excessive conversion and secretion of lactic acid in the TME (a phenomenon known as aerobic glycolysis or “Warburg effect”). Acidification of the TME is further increased by forced aerobic glycolysis in tumor-associated fibroblasts, turning it into a factory for the production of energy-rich metabolites useful for cancer cell proliferation (a phenomenon known as the “reverse Warburg effect”) (13, 14). In this context, alterations in autophagy, as reduction in mitophagy, may favor HCC development due to the increased mitochondrial ROS production consequent to the acceleration of glycolysis. Furthermore, the hypoxic and acidic environments modulate macrophage phenotyping and T-cell cytotoxicity, promoting an immunosuppressive TME (15, 16). Moreover, by the selective degradation of iron-containing macromolecules (e.g., ferritin and mitochondrial components), autophagy is involved in cell death by ferroptosis. Since CD8+ T cells mediate tumor killing also through ferroptosis, autophagy might potentiate this mechanism and sensitize tumor to immunotherapy and radiotherapy (17).

Autophagy also plays a key role in the innate immune response against pathogens, a cellular process named “xenophagy.” Various host factors regulate the replication of the hepatic viruses (i.e., HBV and HCV), which induce radical alterations in the cellular structure activating critical signals that can lead also to autophagy. Recognition of pathogen-associated molecular patterns (PAMPs) is mediated by specific receptors such as TLRs and RIG-I-like receptors (RLRs), which recruit adaptor proteins and various protein kinases involved in the cell transcription and production of type I antiviral interferons (IFN-α/β). Meanwhile, the release of cytokines and chemokines resulting from infected cells activates an inflammatory response, which may also produce extensive tissue damage and sometimes liver infection-associated immunopathies like Non-Alcoholic Fatty Liver (NAFL), steatosis, cirrhosis, and HCC (18). As obligate intracellular parasites, the viruses evolved to hijack host factors and facilitate their replication through several mechanisms able to evade antiviral pathways including autophagy (19). For example, it has been observed that specific proteins of HBV (i.e., X protein) and HCV (i.e., NS4B, NS5A, and NS5B) stimulate autophagy by regulating the expression of genes mediating mitochondrial fragmentation (20–22). At the same time, viruses inhibit the lysosome fusion or impair lysosomal acidification in order to block the physiological function of autophagy and thus ensure membranes for its particle assembly. Moreover, viruses redirect phagosomes to the plasma membrane for the release of viral particles, ensuring pathogen propagation (23, 24). In the same way, by degrading intracellular pathogens and undesirable cytoplasmatic constituents, autophagy can deliver endogenous antigens to MHC molecules, thus allowing antigen presentation to the immune cells and activating an adaptive immune response (12). However, the role of autophagy in HBV- and HCV-related HCC is still widely debated, and further studies are needed to finally understand its function.

Autophagy defects in the liver have been implicated in most liver diseases, which develop following accumulation of harmful products due to the long half-life of hepatocytes (6–12 months) and the considerable number of xenobiotics reaching the liver from the bloodstream and the intestine. As a result, hepatic autophagy is orchestrated by the fluctuation of the body’s nutrient status (e.g., glucose, adipokines, amino acids, and bile acids). Moreover, the circadian rhythm and several hormones (e.g., insulin, glucagon, ghrelin, epinephrine, glial cell line-derived neurotrophic factor, Thyroid-Stimulating Hormone (TSH)) play an important role in the physiological fluctuation of autophagy in the liver, maintaining cellular and metabolic homeostasis. Recently, in this context, intracellular lipid droplet metabolism (e.g., lipophagy) has received much attention, as this mechanism, by regulating the level of β-oxidation to produce ATP, has been related to obesity and insulin resistance, which in turn are strongly associated with the development of Non-Alcoholic Fatty Liver Disease (NAFLD) and non-alcoholic steatohepatitis (NASH) (25). Furthermore, autophagy has been identified as an important mechanism that activates hepatic stellate cells (HSCs) to produce extracellular matrix deposition like collagen involved in liver fibrosis and cirrhosis progression (26). Overall, a reduction of autophagy may act as a tumor-promoting factor by reducing hepatocyte quality and increasing genomic damage, whereas its upregulation may favor the elimination of products originating from metabolic stress and cytotoxic chemotherapy. Furthermore, since autophagy is able to eliminate the mutant p53 tumor suppressor, one of the most frequent mutated protein involved in the proliferation and survival of liver stem cells, a reduction in autophagy favors the maintenance of hepatic stem cells, which play a key role in supporting cancer cells and in promoting tumor recurrence and resistance to therapy (27, 28).

To date, studies targeting autophagy are promising for a potential therapeutic approach in HCC patients, although this approach is challenging due to the high fluctuating differences in cell types. Well-known inhibitors (such as chloroquine, hydroxychloroquine, clarithromycin, or verteporfin) and activators (rapamycin, metformin, temsirolimus, or everolimus) of autophagy are recognized, but they cannot be used in medical practice. New compounds with different targets in the autophagy process are currently investigated to determine their potential to treat HCC and overcome resistance to cancer treatment (29, 30).

### Necrosis

Necrosis is a form of cell death characterized by loss of plasma membrane integrity, culminating in the escape of cell contents into the extracellular space of damaged-associated molecular patterns (DAMPs)/PAMPs and heat shock proteins (HSPs), in contrast to the packaging of cellular contents into apoptotic bodies and release of cytochrome C from mitochondria typical of the apoptosis process. As a consequence, apoptotic bodies occurring in cell death allow for the formation of a “silent” immunological environment, while necrosis induces a strong inflammatory milieu that leads to a high immune response (3, 31, 32).

The biochemical classification of necrosis also included forms of regulated cell death such as necroptosis, ferroptosis, and pyroptosis.

**Figure 2:**
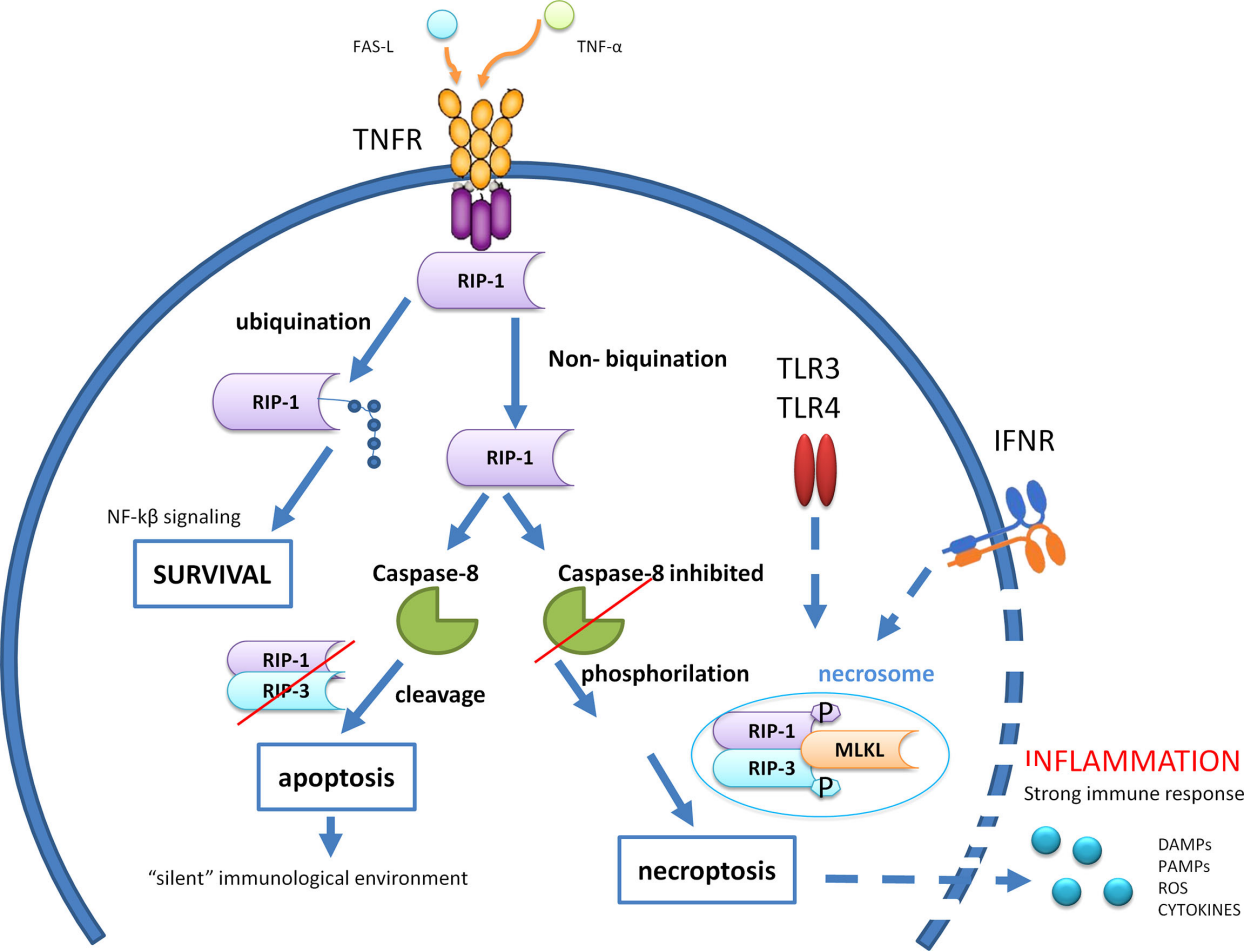
Caspase 8 modulates the crosstalk among cell survival, apoptosis, and necroptosis after engagement of tumor necrosis factor (TNF) death receptors. RIP,receptor-interacting protein; MLKL, Mixed Lineage Kinase Domain Like Pseudokinase; TLR, Toll-like receptor; IFNR, Interferon-gamma receptor; DAMPs, Damage-associated molecular patterns; PAMPs, Pathogen-associated molecular patterns; ROS, Reactive oxygen species; NF-kβ, Nuclear factor kappa-light-chain-enhancer of activated B cells; FAS-L, Fas Ligand; TNF-α, Tumor necrosis factor alfa.

Necroptosis is characterized by a cell lysis occurring when caspase 8 is inhibited. Receptor-interacting protein (RIP)3 kinase is the key regulator of necroptosis that, by interacting with RIP1 and mixed lineage kinase domain-like (MLKL), forms a protein complex termed “necrosome” that triggers necroptosis (**Figure 2**). Indeed, phosphorylation of MLKL by RIP kinases is necessary for the MLKL oligomerization that consents its introduction into and the permeabilization of the plasma membranes and organelles (33). Moreover, RIP3 seems to induce the production of ROS, which in turn at high levels can inhibit cancer cell metastasis; thus, RIP3 expression may also promote antimetastatic outcomes (34). Activation of apoptosis and necroptosis signaling is finely modulated through the function of caspase 8, which inhibits the necrosome *via* inhibition of the RIP1 function **(** **Figure 2** **)**. Cellular receptors triggering necroptosis are TNF death receptors (e.g., TNFRSF1, FAS), TLR4 and TLR3, and cytosolic nucleic acid sensors as RING and STING that induce the production of type-I interferon (IFN-I) and TNF-α that engage TNFR and further induce necroptosis (35). Necroptosis has a key role particularly in the host defense against infectious diseases and tumor cells where components inhibit caspases to arrest the apoptotic machinery, thus resulting in necroptosis as an alternative pathway to overcome apoptosis resistance. There is evidence that necroptosis plays a key role in dysmetabolic liver diseases, in particular, in NASH and cases of tissue damage related to ischemia/reperfusion (36, 37). In addition, although the functional role of necroptosis in HCC development remains to be defined, it has been hypothesized that some chemotherapeutic drugs able to induce necroptosis may overcome the resistance to apoptotic cell death due to, e.g., the loss or inactivation of the p53 protein (34, 38).

Apoptosis is a caspase 8-dependent non-inflammatory form of cell death, whereas necroptosis is a necrotic form of cell death that requires caspase 8 inhibition to achieve RIP1 and RIP3 phosphorylation and production of a functional RIPK3–MLKL signaling (necrosome). Normally, caspase 8 triggers apoptosis by activating caspases, but it also cleaves RIP1 and RIP3, thereby inhibiting necroptosis. Apoptotic bodies occurring in apoptosis allow for the formation of a “silent” immunological environment, while necrosis induces a strong inflammatory milieu by producing and releasing inflammatory mediators (DAMPs, PAMPs, ROS, cytokines) that leads to the recall of immune cells and a high immune response. Ubiquitination of RIP1 both suppresses cell death and promotes cell survival through activation of the NF-κB signaling.

Ferroptosis is a subtype of necrotic cell death determined by the intracellular accumulation of iron that catalyzes lipid peroxidation occurring in neoplastic cells and steatohepatitis (39). Hepatocytes are the primary site of the storage of iron and control the glucose and lipid concentration in the body, resulting in a strict association between liver damage and iron overload (40).

Pyroptosis is a gasdermin-mediated inflammatory response induced by intracellular pathogens and metabolic products, like the ATP released from damaged cell, which activate the inflammasome. The inflammasome is an intracellular multiprotein complex involved in the innate immune response and acts as a platform for the production of inflammatory IL-1β, IL-18, and gasdermin, a specific substrate of caspases 1, 4, 5, and 11 (while apoptosis activates caspase 3). Massive cell death by pyroptosis has been involved in HCC progression where the perpetuating stimulation of inflammation due to, e.g., chronic hepatotropic virus infection or alcohol-mediated liver damage creates a microenvironment that favors NASH and tumor growth (37). Induction of pyroptosis in the liver may also take place from stimuli occurring outside of the liver, e.g., the release of DAMPs due to ischemia/reperfusion of the kidney. Inflammasomes receptors include nucleotide-binding oligomerization domain-like receptor (NLR), IFN-inducible protein (AIM2, IFI16), and pyrin (41).

Overall, the intensity and type of insult decide the type of cell death, while the expression of specific proteins and enzymes (**Table 1**) regulates the process of specific type of death. Liver cancer development has been associated with different risk factors and carcinogenic mechanisms associated with liver cancer (42), leading to a wide tumor heterogeneity, resulting in the definition of diverse behaviors and prognosis of the tumor (43–45). Accordantly, it has been proposed that the consequent type of cell death may favor specific liver regeneration and chromosomic instability leading to different types of liver tumors. Indeed, apoptosis had been reported more associated with HCC differentiation of the malignancy while necroptotic cell death with cholangiocarcinoma development (46, 47). While HCC, the most predominant type of liver tumor, shows a solid, trabecular, and sometimes pseudo glandular growth pattern with a high density of tumor cells originating from hepatocytes, cholangiocarcinoma shows a ductular, papillary, or solid-type structure in a dense stromal tissue, originating from cholangiocytes, and tends to metastasize earlier (47).

The modern approach of immunotherapy in cancer treatment is based on the possibility to boost the host immune response. Cancer cells may induce immunosuppression and resistance to cell lysis by changing the sequence (immunoediting) and expression of the tumor antigens leading to a lower T-cell antigen recognition and by modulating the expression of immune ligand/receptors that induce immune tolerance and immunosuppression (48–53). In this context, modulation of cell death, with effects on immune tumor antigen expression and TME, could be a promising strategy for both tumor treatment and tumor recurrence in long-term immune memory.

Indeed, immunogenic cell death allows the release of specific tumor antigens and pro-inflammatory cytokines, exposes molecules associated with cell damage, DAMPs (49), and modifies the molecules involved in the expression of immune checkpoint, thus reducing immunosuppression and restoring the possible antitumor activity of immune cells.

The main DAMP molecules released during immunogenic cell death primarily depend on endoplasmic reticulum stress activated by ROS and are clustered in groups according to their location (53): calreticulin, HSP70, and HSP90 appear on the cell surface; uric acid, pro-inflammatory cytokines, and high-mobility group box 1 (HMGB1) protein are on the extracellular space; ATP, RNA, and DNA are passively released in the microenvironment following cell death. The release of such DAMPs contributed to the recruitment and maturation of antigen-presenting cells (APCs) in the TME, thus favoring the stimulation of the antitumor immune response, a typical feature of immunogenic cell death. DAMPs mainly act by mediating immunostimulatory effects *via* pattern recognition receptors (PRRs) as the TLRs. In particular, the release of ATP by dying cells enables the recruitment of APC; the APC interaction with calreticulin, HSP70, and HSP90 favors the phagocytosis of dying cells; and finally the presence of HMGB1 and pro-inflammatory cytokines in the extracellular space promotes the maturation of APCs and their cross-presentation capacity for an effective stimulation of antitumor T cells [reviewed in Galluzzi et al. (54)].Of note, locoregional treatments such as radiofrequency ablation with and without adjuvant chemotherapy have been shown to modulate the expression of HSP, resulting in targets for the development of new anticancer therapies as better explained in *Immunological Implications of Locoregional Treatment*, *Surgical Devices and Surgical Trauma*, and *Combination of Different Therapeutic Strategies*.

Numerous studies are ongoing to decipher the complexity of cell death programs to supply key molecular factors to decipher mechanisms sustaining differences in tumor development and modulate cell death under specific conditions. In particular, novel drugs targeting specifically necrosis to redirect the immune response in the TME are under study. Another important research topic is the development of a therapeutic algorithm that takes into consideration the best available treatment by combining the biological behavior of the tumor and the host antitumor immune response.

Particular attention had been paid in this review to reveal the impact of specific surgical interventions on the immune response in HCC.

## Immune Cells and Liver Immunology

The hepatic environment is unique in that its sinusoidal cellular structure favors the extravasation of cells, including neoplastic ones, and for its specific ability to filter an enormous quantity of immune antigens from nutrients and bacteria from the gastrointestinal tract while still being tolerogenic to these molecules (55). It has been calculated that 30% of all circulating blood mass passes through the liver every minute; so, there is a continuous exchange of immunological information from various body districts.

Different cell populations are involved in the regulation of the immune homeostasis in the liver.

Kupffer cells, resident macrophages invading the Disse space, are the largest population of hepatic immune cells (28). There are two Kupffer subpopulations: the M1 CD68+ subset with phagocytic capacity and producing nitric oxide (NO) and ROS and the M2 CD11b+ subgroup that produces cytokines, like IL-10 and IL-12. Both subsets can modulate the growth of hepatocytes and produce DNA damage. In response to liver lesion, they produce an elevated level of chemokines like CCL2 and CXCL10 that recruit immune cells in the liver as needed for liver regeneration.

Dendritic cells are in the subcapsular space to protect the liver from bacteria, viruses, toxins, and metastasizing neoplastic cells arriving from the peritoneal cavity by capturing circulating antigens and presenting them to T cells (56). However, tumor cells produce chemokines and cytokines as CCL22 and TGF-β to recruit regulatory T cells (Treg) that impair this immune response, favoring HCC growth (57).

The function of granulocytes, neutrophils, and eosinophils arises from the gradient of chemokines that accumulate in blood vessels during liver damage, and they gather in the parenchyma of the liver without the need for adhesion molecules (58).

Lymphocytes activated in the liver are up to 65% cytolytic natural killer (NK) cells with a role against HCC and chronic hepatic infections (59), whereas B lymphocytes during an infection are organized in intraportal follicles surrounded by dendritic cells and T cells similar to the functional structure of the germinal center observed in lymph nodes, suggesting a similar function (production of IgM and memory B cells) in the liver (60).

The balance between the immunogenicity of the tumor cells and the function of the immune cells present in the microenvironment decides the host immune response against the tumor. Accumulation of genetic mutations and alterations of cell signaling in cancer cells influence tumor immunogenicity, including the expression of new antigens [tumor-associated antigens (TAAs) and tumor-specific antigens (TSAs)]. However, environmental factors, e.g., pathogens, or age and stress, can affect the immune response to cancer, also causing resistance to immunotherapy, as the inhibition of immune checkpoints. During the development of HCC, cells expressing a high level of TAA could be removed by the host’s immune system (61); accordingly, the activity of CD8+ T cells against tumor cells was found to be higher in early-stage HCC than that in cases with an advanced stage. By the same way, it is now clear that a strong chronic antigen stimulation may induce immune cell anergy, exhaustion, and senescence, thus contributing to the failure to eradicate the tumor and bringing to an immunosuppressive microenvironment with anergic T cell in advanced-stage malignancy (62). Thus, tumor cell subclones showing few common driver mutations may become prevalent during tumor growth, contributing to a highly heterogeneous HCC population (63). In line with this model, tumors with mismatch repair deficiency, carrying a high number of passenger mutations, were found to usually have the best response to immunotherapies (64). To increase the benefit of this therapeutic strategy, the TME should be changed to restore the host antitumor immunity. Based on the evaluation of immune cell types and the ratio present in the microenvironment further to the usual histological/molecular classification of HCC, a classification based on differences in the immunological structure of the microenvironment in HCC has been proposed (65). Indeed, by using multiplex immunohistochemistry enables a direct analysis of the immune microenvironment with histological information, HCC can be classified into three immunosubtypes such as Immune-high, Immune-mid, and Immune-low subtypes. Of note, the proposed immune subtypes displayed different prognoses and also perform well in predicting strategies for improving the efficacy of HCC immunotherapy, highlighting the necessity to take in consideration methylation and miRNAs as modulators of the complex immune response.

## Immunological Implications of Hepatocellular Carcinoma Recurrence After Transplantation

As previously mentioned, the different types of cell death induce local inflammation with various intensities. Indeed, apoptosis usually evokes a minor inflammatory response compared to necrosis, necroptosis, and pyroptosis, which are characterized by a strong inflammatory environment due to the release of DAMPs. The inflammatory milieu can favor the recruitment of immune cells able to recognize and kill tumor cells but can also promote the release of tumorigenic factors (5). Thus, inflammation can be either beneficial or detrimental to the liver. Indeed, mild inflammatory responses can favor tissue repair and reestablishment of the homeostasis. On the contrary, excessive inflammation may imply a loss of hepatocytes, causing an irreversible liver damage with consequent fibrosis and possible carcinogenesis (66). Thus, the monitoring of the inflammation intensity, also at a systemic level, could be pivotal after local treatment for prognosis purposes. It is well accepted that systemic immune deregulation in inflamed liver could be evaluated by the Systemic Immune-Inflammation Index (SII), which is calculated by (N × P)/L, where N, P, and L represent neutrophil counts, platelet counts, and lymphocyte counts, respectively. The inflammatory pathway is associated with an increase of neutrophils that release elevated levels of inflammatory cytokines (67) and an increase of platelets that favor neoangiogenesis by the release of vascular endothelial growth factor (VEGF) and the activation and proliferation of lymphocytes (68). The test is easy to perform, but it must take into consideration that SII parameters are extremely influenced by many causes (e.g., gastrointestinal bleeding) also independent of HCC. Since inflammatory status in the liver is strictly related to cell death and immune alteration present in the TME as reported in earlier sections, SII has been proposed also to evaluate the immune perturbation happening locally in HCC.

The most important result has been obtained in the setting of liver transplantation where it has been observed that preoperative HCC-related immunosuppression may persist after liver transplantation, and this reduces not only the incidence of acute rejection (69, 70) but also HCC recurrence (71). In particular, a high level of neutrophils as a marker of necrotic cell death and high inflammatory response is associated with a high risk of HCC recurrence after transplantation (72).

In addition, the decision to treat patients with an inadequate response can be challenging, since the treatment of acute rejection with steroid that modulates the host immune response was found to increase the risk of HCC recurrence 18-fold (73). By converse, the inhibitors of the mammalian target of rapamycin (mTORi), e.g., everolimus and sirolimus, had shown a favorable effect in reducing the incidence of HCC recurrence compared to standard immunosuppressors (70, 74).

Today, transplantation research programs engage effort toward a scrupulous use of immunosuppressive strategies to protect the graft by keeping the host sufficiently competent in treating infections and neoplasms. The use of novel immune strategies, such as immune checkpoint inhibitors (ICIs), receptor T-cell engineering, and vaccines, other than the main immunosuppressants, is promising to ensure the therapeutic aim in the future.

## Immunological Implications of Locoregional Treatment

Locoregional therapies for HCC can be offered with curative intent, with palliative intent when other radical options cannot be considered due to the extent or the severity of the disease, or with downstaging intent as a bridge for transplantation.

Local ablative strategies with curative intent are used to treat small tumors (<3 cm) in favorable positions, as tumors >3 cm are commonly prone to arterial embolization. Other strategies such as percutaneous ethanol injection (PEI), cryoablation, high-intensity focused ultrasound (HIFU), radioembolization [transarterial radioembolization (TARE)], and stereotactic body radiotherapy (SBRT) can also be employed as palliative or downstaging treatments (75–77).

Locoregional treatments induce different cellular damages depending on the strategy used: microwave and radiofrequencies cause thermal insults leading to necrosis, denaturation, and coagulation of tumor proteins, while cryoablation causes damage through formation of ice microcrystals in the intracellular organelles and the rupture of the cell membrane. Damage then leads to an inflammatory status with the appearance of pain, fever, vasodilation, and an increase of vascular permeability, while the necrotic cell debris may be a source of tumor antigens available for the stimulation of the immune system (78–80).

Temperature can influence the immune response, in particular, CD8+ cytotoxic T-cell proliferation and function were enhanced by using high temperature for short times probably through the alteration of the immune cell membrane (81–83). Moreover, it was shown that an insufficient thermoablative treatment may favor the growth and spread of HCC by favoring portal invasion and poor cell differentiation. Indeed, residual neoplastic cells can be stimulated to grow by neoangiogenetic factors like Hypoxia-inducible factor-1 (HIF-1) alpha and VEGF A released as a consequence of tissue hypoxia (84). Additionally, the protumorigenic environment resulting from a sublethal ablation may be reflected also at a systemic level due to the diffusion of cytokines in the serum. Indeed, experimental studies demonstrated that the growth of distant cancers was found related to the different parameters of ablation performed in the liver (85–89). The key immune modulators produced by damaged liver tissue after ablation are the HSPs, which are expressed as a defense against hyperthermia and may serve as a prognostic marker of ablation, although warranting further tests in clinical settings (90). Recent reports highlight the perspectives of targeting HSP as a future tool for cancer treatment, e.g., liposomes loaded with quercetin, an HSP inhibitor (91–93).

Thus, with advances in computational techniques and digital graphic approaches, locoregional ablation using hyperthermia has been developed to treat HCC as an effective approach and by the same way to reduce the immunosuppressive milieu of the microenvironment, also by developing adjuvant protocols able to reduce the expression of HSPs.

The same way that ablation may release specific tumor antigens from necrotic debris, experimental studies have been focused on the identification of immunogenic potential antigen(s) induced by ablation. Ficolin-3, a complement-activating protein produced by the *FCN3* gene, has been one of the most promising both as a prognostic marker for ablation treatment efficacy in HCC and a potential target for immunotherapy (80, 94).

## Surgical Devices and Surgical Trauma

Surgical device for HCC refers to the tissue cutting and coagulation of the parenchyma using high energy leading to tissue necrosis and warming hemostasis. The two most common energy devices used worldwide are bipolar vessel sealing systems and ultrasound (95). A third device combines both ultrasonically generated thermal energy and advanced bipolar energy to dissect and scrap the tissue with minimal thermal diffusion that produces highly localized heat compared to the other energy devices (more than 200°C compared to 100°C for electrosurgery).

The harmonic ultrasound scalpel, by using vibrational forces, dissects the tissue and seals the vessels generating water vapor from the cells, while the electric scalpel, by using electrical energy to smoke the tissue, produces burned debris and harmful chemicals (e.g., hydrogen cyanide, acetylene, and butadiene) that are potentially toxic. The limited thermal heat generated by the ultrasound scalpel also minimized the zone of thermal damage (does not exceed 80°C) compared to the electric scalpel (up to 500°C). Huang et al. (96) clearly proved a benefit in terms of disease-free survival in patients receiving ablation treatment with radiofrequency. The observed benefit appeared to be widely associated with a local and systemic immune regulation. They showed the infiltration and activation of the adaptive immune cells (APCs, macrophages, NK cells) in the tumor area with proliferation, activation, and survival of memory and CD8+ T cells and a reduction of inhibitory cytokines and an increase of antitumor cytokines in the peripheral blood. It is believed that the induced systemic antitumor immunity overcomes the challenges of micrometastases, which often escape tissue destruction. Then, in a mouse model, authors proved the effect of the tumor immune environment after ablation on distant tumors by demonstrating that the tumor-specific immune response induced by the combination of ablation and immunotherapy [anti-Programmed Cell Death Protein 1 (PD-1)] was stronger than anti-immune (anti-PD-1) or ablation alone (97).

Surgery, including liver transplantation, is the most efficient treatment for patients with >3 cm HCC lesion. However, the indication for transplantation is restricted to a few patients.

Surgical resection was found to generate an acute-phase response together with an extensive immune-inflammatory response and a strong immune-suppressive status that was not seen in less invasive procedures (98). Cytokine imbalance between inflammatory (i.e., IL-6, IL-8, IL-10, CCL2) and anti-inflammatory (IFN-γ) and a suppressed cellular immunity (NK, total lymphocytes, and dendritic cells) were observed after surgery, while an elevated neutrophil/lymphocyte ratio may persist for up to 6 months after surgery. In turn, the inflammatory status in the postoperative period influenced the HCC prognosis and may favor the formation of a premetastatic niche. Moreover, the hypoxia generated in the liver by surgery causes a stimulation of the parenchymal cells, which take part in angiogenesis and wound repair of the liver, but also causes a de-differentiation of the residual tumor cells with high metastatic ability and the release of inflammatory mediators.

Thus, it was reasoned that perioperative therapy aimed at minimizing the consequences of surgery-induced tissue hypoxia and inflammation could be beneficial to reduce tumor recurrence.

## Combination of Different Therapeutic Strategies

There is growing evidence that surgical resection/ablation of the HCC lesion disrupts the host’s immune system, creating an immunosuppressive window for the expansion and escape of occult metastases and had a prognostic effect on disease-free survival time.

To reduce this window, the introduction of drugs able to modulate the systemic immune response in combination with thermal ablation, such as the combination of propranolol (β-adrenergic inhibitor) and etodolac (COX-2 inhibitor) to inhibit the release of surgery-induced catecholamines and prostaglandins, has been evaluated. Other approaches proposed are the use of prostaglandin inhibitors in combination with the PD-1 inhibitors to restore the cytolytic CD8+ T-cell function after surgery, the inactivation of the platelets to reduce the risk of prothrombotic events and cancer metastasis, and the administration of dendritic/virus-based vaccination before perioperative surgical resection (99–101).

A new promising approach for HCC treatment is the irreversible electrochemotherapy that is a non-thermal ablative method based on the delivery of short high-power electric pulses between two electrodes inducing pores across the cell membrane and consequent apoptosis (102). This strategy combines a single dose of either bleomycin or cisplatin with irreversible electroporation, causing a significant increase in the cytotoxic effect. Treatment with irreversible electrochemotherapy of 75 HCC nodules in 58 patients who were not eligible for thermal ablation provided complete ablation rates of 77.3%, 89.3%, and 92% after one, two, and three procedures, respectively (103). In the treated patients, the 12-month overall tumor progression-free survival was 70%. Electrochemotherapy, similarly to other ablative treatments, likely elicits a local immune response against HCC tumor antigens and may boost adjuvant immunotherapy with ICIs or cytokines to achieve a long-lasting anticancer response (104). Noteworthily, the promising introduction of ICI in the treatment of advanced HCC showed a higher efficacy in case of viral etiologies compared to non-viral-related tumors by virtue of the increased immunogenicity of virus-derived neopeptides. Conversely, NASH-related HCC appeared less responsive to ICIs due to the presence of a dysfunctional infiltration of CD8+PD1+ T cells probably induced by the obesity-caused metabolic changes within the TME [reviewed in Haber et al. (2)]. Finally, several other immunotherapeutic treatments have been proposed as adjuvant therapy in HCC after local treatment, bringing different results (2). Immunotherapy with cytokine-induced killer (CIK) cells improved survival of HCC patients already treated with percutaneous ethanol injection, radiofrequency ablation, or surgical resection without worsening adverse events (105). On the contrary, the adjuvant employment of IFN-α after resection did not demonstrate a survival advantage (106, 107).

## Discussion

The locoregional thermal ablative treatment of HCC causes many local and systemic phenomena able to change the response to treatment and the patient’s prognosis; different strategies that affect the modulation of T lymphocytes could represent a promising approach.

A difference in the immune response according to the technique used for ablation and surgical resection has been proven independently of the patient’s genetic background. The treatment of HCC is currently based on the size, location, and severity of the disease, but as evaluated in this review, other variables, such as the immune response, could be also taken in consideration in the future, as they can imply a better response and survival prognosis. Although a diagnostic therapeutic diagram including immune variables has not yet been elaborated, several therapeutic strategies combining resection and immune modulation, associated with the type of tumor cell death, are currently under evaluation for advanced-stage HCC. At the same time, the pathogenesis underlying the different HCC subtypes could better define which type of immunosuppressive microenvironment is associated with disease recurrence, thus favoring the identification of the best combination treatment among all surgical and pharmacological options. The systemic inflammatory response could be also an important target for further studies as a potential source of key prognostic factors and targets for adjuvant treatments.

The study of the modulation of the immune response using thermal stimuli in combination with drugs and/or surgical procedures has just begun. However, the debate on this issue with the proposal of possible personalized treatments that combine the specific immune status of each patient to obtain a better therapeutic efficacy seems promising.

## Author Contributions

VD and ARS conceived the study and drafted the manuscript. ARG, EM, VR, MT, AZ, and AU reviewed the entire manuscript and coordinated the work to provide a comprehensive overview of the multiple aspects taken into account in the review. All authors read and approved the final manuscript.

## Funding

This work was supported by the Italian Ministry of Health (Ricerca Corrente).

## Conflict of Interest

The authors declare that the research was conducted in the absence of any commercial or financial relationships that could be construed as a potential conflict of interest.

## Publisher’s Note

All claims expressed in this article are solely those of the authors and do not necessarily represent those of their affiliated organizations, or those of the publisher, the editors and the reviewers. Any product that may be evaluated in this article, or claim that may be made by its manufacturer, is not guaranteed or endorsed by the publisher.
